# Evolution in Dentistry

**Published:** 1890-06

**Authors:** J. A. Robinson


### THE DENTAL REGISTER.

VOL. XLIV.] JUNE, 1890. [No. 6.

### COMMUNICATIONS.

### Evolution in Dentistry.

BY J. A. ROBINSON, D. D. S.

Evolution is growth from the infinitesimal beginning of all life, the protoplasm or first form to the eternal or everlasting life. At this beautiful spring time, when everything is rejuvenating or springing forth into life in the physical world, it is worth while to pause and reflect on the great first cause of all life, progress and improvement. The maples and the elms drop their seeds upon the earth, which the frost and the snows have prepared for their reception, and by a providential reaction of the elements reproduce in beautiful order the counterpart of themselves to act and re-act and grow until the end of time, but the *life*, the *being*, the first great cause, was from Eternity.

We have *being* and *existence*; the being before the existence, and the existence because of the *Being*. Society is a correspondence of nature; the march of improvement, the growth of our civilization—if we read history rightly—is thoroughly illustrated by the seasons of the year and the animal and vegetable world.

We are like corn—the world is but a field—
If we are true, a thousand fold we yield.
Go search a field of corn all through and through,
Alas! the perfect ears are very few.
The fruit, as it appears upon the ears,
Reminds us of our youth and wane of years;
The early kernels are so undefined
They seem to represent the youthful mind;
The center of the ear is round and fair—
Strong middle life is represented there.

The wrinkled, shriveled seeds that end the ears,
Display as fully our declining years.
So when the whole is full, and ripe, and round,
Like age it bends its head toward the ground.
We live and grow upon the corn at last,
As nations grow upon the ages past.
If in this life we've good or evil done,
The fruit thereof will shake like Lebanon.

As is society and man, so are professions. Nature is our teacher. The thought of liberty was undoubtedly suggested to the slave by his unequal lot in life as he beheld the birds flying to more congenial climes, soaring above his head in pursuit of fairer and happier abodes in another clime and land.

Think of the years dentistry has been journeying to the land of promise. It was not till 1857 that cohesive foil had the confidence of our best operators. I think Dr. Atkinson must have the credit of the serrated pointed or bifurcated plugger. In 1854 I recollect making a double point to my graver pointed instrument, and Dr. Atkinson suggested a *foot plugger* made in the same manner. This gave an impetus to cohesive foil, which has grown so fast into a necessity in perfect dental practice. Ten years afterward some good dentists in Boston abandoned cohesive foil and went back to soft foil exclusively for saving teeth, but the cohesive foil went on all the same. Some time about 1856 an old gentleman came into the office in Cleveland with oxyphosphate as a filling for teeth. His name was Tyndale, I think, and that was the first I ever heard of a material that now enters as largely into dental practice, and is as indispensable, as anything we have in saving teeth.

It has taken years to convince the incredulous that pure gold is itself cohesive, but that time is fast passing or has fully passed away. It was left to Williams to manufacture and fully perfect the corrugated and cohesive foils, as it was also left for Stearns to succeed in making for himself an artificial vellum for congenital fissure of the palate, which was only made possible by the use of flexible rubber, as it has also taken a hundred years of evolution in electricity and a Franklin and Morse and Field to perfect the telegraph and Edison the telephone of the present time. When the thought is once born and fed by loving hands it is sure to grow.

The advent of a new thought or a new process in a machine is almost always a necessity—some obstacle needs to be overcome. The imperfections of our operations have somehow forced upon us our improvements. The dental engine, one of the most useful of all our appliances, was originally made as a labor-saving machine for shearing sheep and was modified for the use of the dentist. It did not serve the purpose for which it was first intended, but the genius of the dentist has made it a perfect labor-saving thing, and no one can do without it. The burs of the engine that make it useful are as old as dentistry. I made hand burs when a boy, and have one in my possession to-day that I made more than fifty years ago. It is like the ferns we see to-day which belong to the tertiary age, and remind us of ages that have passed away. The testimony of the rocks show us there were three successive grades of animals and plants during that age, and the ferns have survived through the entire length of time. It is not the *one* new thing that has carried dentistry to its present efficient position, but the combination of forces applied with thought and power that has elevated us to our present station; the mallet and the gold, the teeth, and even the dental chair have assisted and made possible the evolution we find to-day.

Our alliance with the medical profession has given us great advantages in education, and also entailed upon us a load like that on the back of Bunyan's Pilgrim—that is, a load too burdensome for this close of the nineteenth century. The atmosphere has been somewhat darkened and the sun somewhat obscured, but the law of evolution and growth is always active and upward and there will always be a survival of that which is fittest to survive. I allude to the code of ethics which has become a dead letter in the hearts of the broadest members in both professions, and which ought to be relegated to that class of animals that are destitute of a vertebrate.

The resolution for expulsion of Dr. John S. Chance from the Ohio State Dental Association, and of Dr. Land, of the Michigan Association, for advertising, came near producing a rupture between the members that could not be healed, and we shall find that it is unwise for any association to run against the spirit of our civilization. Every organized body of workers is made strong by what it *includes* rather than by what it *excludes*, in a republic. It is safest and best for members of every association to obey wholesome laws in the societies where they belong, but if the laws do not correspond to the generation in which we live they ought to be modified or abolished.

There is something more to be done if we would grow into a noble profession than filling and extracting teeth; we must be a band of brothers. The antiseptic influence of *manliness* must be thoroughly impressed and exercised, and education is the only real antiseptic influence all over the world, but we can not educate persons by expelling them from our society. Evolution in society necessarily involves change in methods of doing business and the decay of old ways—this is inevitable if we make progress.

The Episcopal Church, which is the most conservative Protestant body in the world, has been struggling for forty years to amend the thirty-nine articles of faith and belief, and now the Presbyterians are doing the same thing; and there is no more reason why a profession scarcely fifty years old should continue to worship an old "code of ethics," and that borrowed from the medical code, which, next to the church, is a most conservative body and entirely unfit for the present needs of the dental profession because it leads to dishonesty in methods of advertising, by an *indirect* way of paying reporters of newspapers to dog their footsteps and note every trifling incident in their daily lives and to parade their names and doings before the public. We must work on in hope, if we expect to realize our desires of reform and growth. The differentiations and growth in human life, like the plant life, never work on parallel lines, but after all are striving toward the higher good as plant life grows toward the sun for light and heat.

---

BOILS and felons are said to be aborted in twenty-four hours by the application of a thick layer of ointment of nitrate of mercury, covering the whole with adhesive plaster.

